# Insomniacs show greater prefrontal activation during verbal fluency task compared to non-insomniacs: a functional near-infrared spectroscopy investigation of depression in patients

**DOI:** 10.1186/s12888-023-04694-z

**Published:** 2023-03-30

**Authors:** HuaSen Xu, YuXing Wang, Yi Ming Wang, YaQi Cao, PeiFan Li, YongXue Hu, GuangYuan Xia

**Affiliations:** Department of psychiatry, Affliated Hospital to Guizhou Medical University, Guizhou, China

**Keywords:** Major depressive disorder, Insomnia, Prefrontal cortex, Functional, Near-infrared spectroscopy, Verbal fluency task

## Abstract

**Background:**

Previous studies have shown that insomnia affects human prefrontal function and that there are specific patterns of brain activation to counteract sleep and improve cognition. However, the effects of insomnia on the prefrontal cortex of MDD (major depressive disorder) patients and the patterns of activation to counteract sleep in MDD patients remain unclear. The aim of this study is to examine this using fNIRS (functional near-infrared spectroscopy).

**Methods:**

Eighty depressed patients and 44 healthy controls were recruited for this study. fNIRS was used to assess changes in the concentration of oxygenated hemoglobin ([oxy-Hb]) in the prefrontal cortex of all participants during the VFT (verbal fluency test) and to record the number of words created to assess cognitive ability. The Pittsburgh Sleep Quality Index was used to assess sleep quality, and the Hamilton Rating Scale for Depression (24-item) and Hamilton Rating Scale for Anxiety (14-item) were used to assess the severity of depression and anxiety.

**Results:**

When comparing patients, the healthy control group had significantly higher [oxy-Hb] values in the bilateral prefrontal cortex during VFT than the MDD group. In the MDD group, the [oxy-Hb] values in all brain regions except the right DLPFC were significantly higher in the group with insomnia than in the group without insomnia, but their VFT performance was significantly lower than in the group without insomnia and the healthy group. PSQI scores were positively correlated with [oxy-Hb] values in some left-brain regions, whereas HAMD and HAMA scores were not correlated with [oxy-Hb] values.

**Conclusion:**

The PFC was significantly less active during VFT in those with MDD than in healthy controls. All brain regions, except the right DLPFC, were significantly more active in MDD patients with insomnia than in those without insomnia, suggesting that sleep quality needs to be an important indicator in fNIRS screening. In addition, there was a positive correlation between the severity of insomnia in the left VLPFC and the level of activation, suggesting a role for the left brain region in the neurophysiology of overcoming sleepiness in MDD patients. these findings may provide new ideas for the treatment of MDD patients in the future.

**Trial registration:**

Our experiment was registered in the China Clinical Trial Registry (registration number ChiCTR2200065622) on November 10.( The first patient was recruited in 10/11/2022.)

## Background

Undoubtedly one of the most prevalent mental illnesses is major depressive disorder(MDD). More than 300 million people worldwide are affected by it [1]. The inability to fall asleep is one of the most common symptoms of MDD [2]. When depressed, 67% to 84% of adults report having trouble falling asleep or staying asleep [3–5]. According to up to 88% of MDD patients who report subjective sleep issues, they have trouble falling asleep [6]. People who struggle with depression and sleep problems are more likely to experience severe symptoms and have difficulty recovering [7]. However, little is still known about how depression and lack of sleep affect the brain, particularly how insomnia affects the brains of patients with depression.

Recent advances in neuroimaging technology have made it possible to gradually observe how people with major depressive disorder and sleep issues' brains function. According to functional neuroimaging studies, people with major depressive disorder and sleep issues have prefrontal cortex issues, which may be connected to their clinical symptoms(including attention, memory, and executive function) [8–11]. By monitoring changes in oxygenated Hb [oxy-Hb] and deoxygenated Hb [deoxy-Hb] concentrations in the brain cortex, functioning near-infrared spectroscopy is a non-invasive neuroimaging technique that can be easily performed in a natural setting and used to help people with psychiatric disorders [12].

The verbal fluency task is employed as a psychological assessment in many functional near infrared spectroscopy (fNIRS) studies. In a short period of time, people try to produce as many words as they can [13]. The verbal fluency task (VFT) primarily assesses verbal and executive control abilities, which are connected to basic neurocognitive functions like working memory, motivation, and attention [14]. Numerous studies [15, 16] have shown that prefrontal cortex (PFC) dysfunctions are associated with neurocognitive issues in a variety of psychiatric disorders. It has been used in conjunction with fNIRS to examine the PFC use of individuals with and without mental illnesses.

Prefrontal cortex activity during the VFT is lower in people with MDD than in healthy individuals, according to earlier fNIRS research [17]. Prefrontal activation also changes when healthy people do not get enough sleep, either increasing or decreasing [18] or both [19]. However, it remains unclear how insomnia affects the activation of the prefrontal cortex in patients with MDD. Motoyasu Honma et al. found that activation of the right prefrontal cortex (but not the left prefrontal cortex) was positively correlated with alertness in tests of alertness in healthy subjects. Therefore, they suggest that activation of the right prefrontal cortex may help healthy individuals or those with short-term insomnia to overcome sleepiness and provide sufficient activity to meet the cognitive demands of higher cognitive tasks [19]. According to Sun et al., oxy-Hb changes in the left prefrontal cortex of some patients with chronic insomnia tend to increase progressively with decreasing sleep quality [11]. It is currently unknown whether depressed people overcome sleepiness in the same way as healthy people and people with chronic insomnia.

Therefore, we designed studies to examine the brain activity of insomniac and non-insomniac MDD patients and used it to analyze the activation patterns of MDD patients against sleepiness. We anticipated that people with MDD would behave worse during the VFT than healthy individuals and would have less prefrontal cortex activity. Furthermore, depression patients suffering from insomnia have poorer activation compared to patients without insomnia. Patients with depression may have different anti sleep patterns from normal people and chronic insomniacs.

## Materials and methods

### Participants

According to the criteria outlined in the fifth edition of the Diagnostic and Statistical Manual of Mental Disorders (DSM-5), patients were recruited by doctors in the outpatient department of Guizhou Medical University Hospital who conducted inquiries into the patient's condition, combined with scale scores and medical history provided by their families. Eighty patients between the ages of 18 and 53, who were identified as having MDD with scores ranging from 16 to 49 on the HAMD-24, the Hamilton Rating Scale for Depression, were included in the study according to the criteria of the DSM-5. Subsequently, the MDD patients were divided into two groups: those without insomnia and those with insomnia. For patients with insomnia, they needed to experience at least one of the following symptoms three or more times per week: (i) difficulty falling asleep within 30 min; (ii) waking up and being unable to fall back asleep within 30 min; (iii) sleeping less than 6 h; or (iv) sleeping less than 85% of the time. There were two groups of MDD patients without insomnia: those who reported experiencing one or more of the above symptoms less than three times a week, and those who did not report experiencing any of these symptoms at all [20]. Only right-handed patients were included in the study. We only included participants who had discontinued the use of any medication three months or more prior to the fNIRS data collection in order to eliminate the effects of drug use.

As healthy controls (HCs), 44 healthy individuals from the local population were selected to match the patients in terms of age, right-hand dominance, educational degree, and gender. The controls did not have MDD or insomnia, and their ages ranged from 21 to 50. They were excluded for the following reasons: (i) severe and unstable physical conditions, (ii) use of antidepressants, sedatives, hypnotics, or other drugs within three months prior to enrollment, (iii) presence of additional mental disorders, (iv) sleep disorders such as restless leg syndrome, narcolepsy, and obstructive sleep apnea, and (v) pregnancy or nursing. All participants in this study provided written consent prior to beginning.

### Clinical assessment

HAMD-24 consists of 24 symptom-based items (including 7 factor structures for anxiety/somatization, weight loss, cognitive disorders, daytime changes, block, sleep disorders, and feelings of hopelessness). with a total score range of 0 to 76 points and item scores ranging from 0 to 4. It is considered mild depression if the total HAMD-24 score falls between 8 and 19, moderate-severe depression if the score is between 20 and 35, and severe depression if the score exceeds 35 [21, 22]. In this study, two versions of HAMD were prepared to exclude the influence of other depressive symptoms on the results. In addition to the HAMD-24 introduced above, we also produced HAMD without sleep disorders, i.e. HAMD containing the other six items except sleep disorders (hereinafter referred to as HAMD without sleep disorders).

Anxiety symptoms are assessed in detail using the Hamilton Anxiety Inventory (HAMA) [23], which is based on clinical interviews with individuals experiencing anxiety. A total score of ≥ 29 indicates severe anxiety, a score of ≥ 21 indicates significant anxiety, and a score of ≥ 14 indicates anxiety. A score of less than 7 indicates the absence of anxiety symptoms. Subscales for "somatic anxiety" and "psychological anxiety" can also be calculated in addition to the total score. Higher scores indicate a more severe condition.

The Pittsburgh Sleep Quality Index (PSQI) was developed in 1989 to identify individuals with "good" and "poor" sleep quality in a consistent and reliable manner. It is a self-administered questionnaire that assesses various aspects of sleep quality over the past month. The PSQI is currently the most commonly used method for evaluating sleep quality [24].

Three different tests were used in this study to measure depression, anxiety, and sleep quality. The HAMD was used to measure depression, the HAMA was used to measure anxiety, and the PSQI was used to measure sleep quality.

### Activation task (Verbal Fluency Task)

**Fig. 1:**
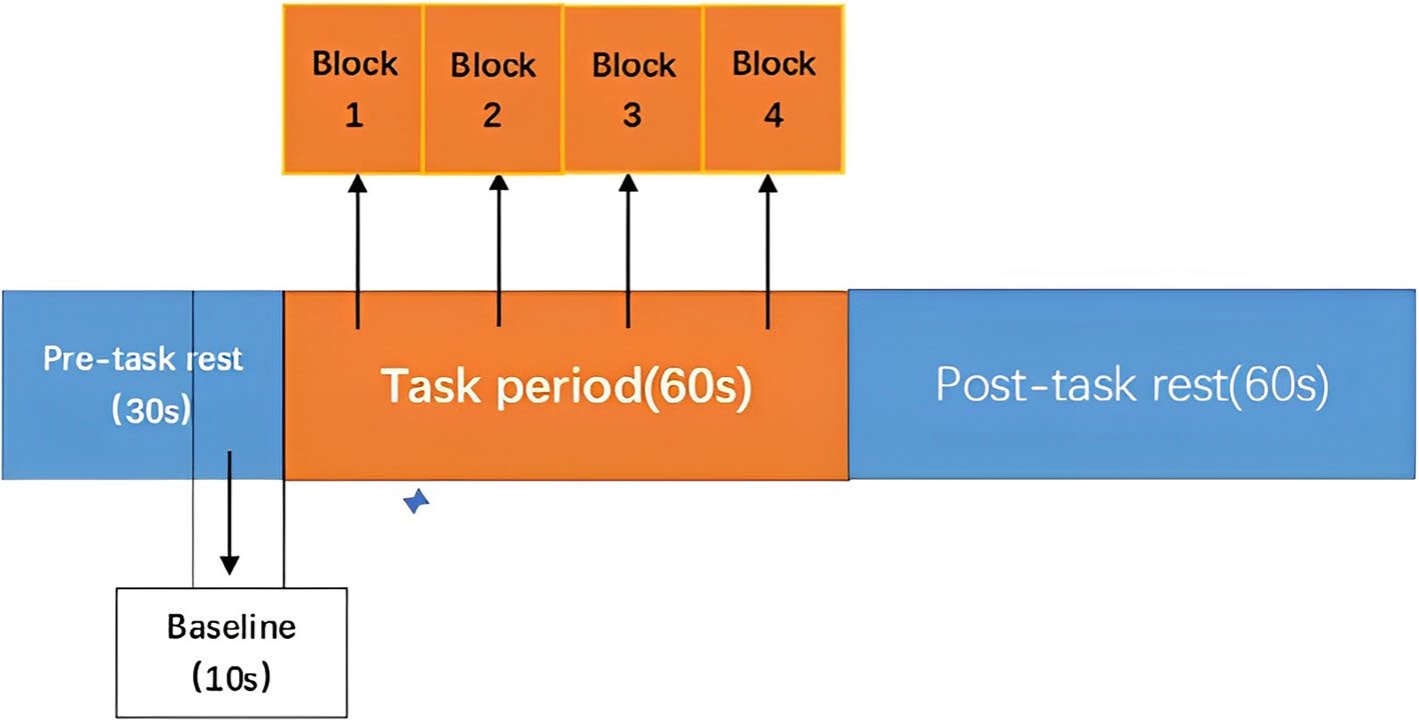
The VFT protocol used for near-infrared spectroscopy. Each trial consisted of a 30 s pre-task rest period, a 60 s task period subdivided into four 15 s blocks and finally a 60 s post-task rest period

In this study, the Chinese phonetic Verbal Fluency Test (VFT) developed by Quan et al. was used to assess cognitive function [25]. Each trial included a 30-s break prior to the task, a 60-s break during the task, and a 60-s break following the task for Chinese participants. The fNIRS device instructed the participants to count aloud a specific number both during and after the task. We divided the 60 s task cycle into four 15 s blocks. The four Chinese syllables "Shang" (上), "Shi" (时), "Shuo" (说), and "Jia" (家) which sound like the words 'up,' 'time,' 'speak,' and 'home,' respectively, are spoken to the subjects once every 15 s. Participants were instructed to generate as many words as possible using these syllable. Everyone received the same syllable cue, and their words were said in the same order. We gave everyone the opportunity to practice so that they could demonstrate their understanding of the material before the actual test. To make sure they were doing what they could to help with the assessment, participants were asked before the study whether they had enough energy to complete the assessment and their subsequent performance on the entire task was observed. Participants with poor energy and distracted attention were excluded from the study. As a final indicator of each subject's cognitive abilities, the number of words they each say will be recorded [26, 27] (Fig. 1).

### NIRS Measurement

In accordance with modified Beer-Lambert law, Twenty-six multichannel fNIRS instruments (Wuhan Union Medical Technology Co., Wuhan, China) measure [oxy-Hb] and [deoxy-Hb] concentrations in the cerebral cortex using infrared light at two wavelengths (670 nm and 830 nm) [28]. [Deoxy-Hb] and [oxy-Hb] can be distinguished by absorbing infrared light using dual-wavelength laser diodes [29]. The sampling frequency was set at 20 Hz. The probe is a cap based on a standard human brain with 16 light sources and 7 light detectors. The space between each pair of light sources and detectors is fixed at 3 cm. A channel is the region between a pair of source probes and a pair of detector probes where measurements are made. So, when we placed the probe set in the participant's prefrontal region via the International 10–20 EEG electrode placement system (the lowest probe was placed along the Fp1-Fp2 line), all 26 channels in the PFC could therefore display different waveforms for [oxy-Hb] and [deoxy-Hb].

**Fig. 2:**
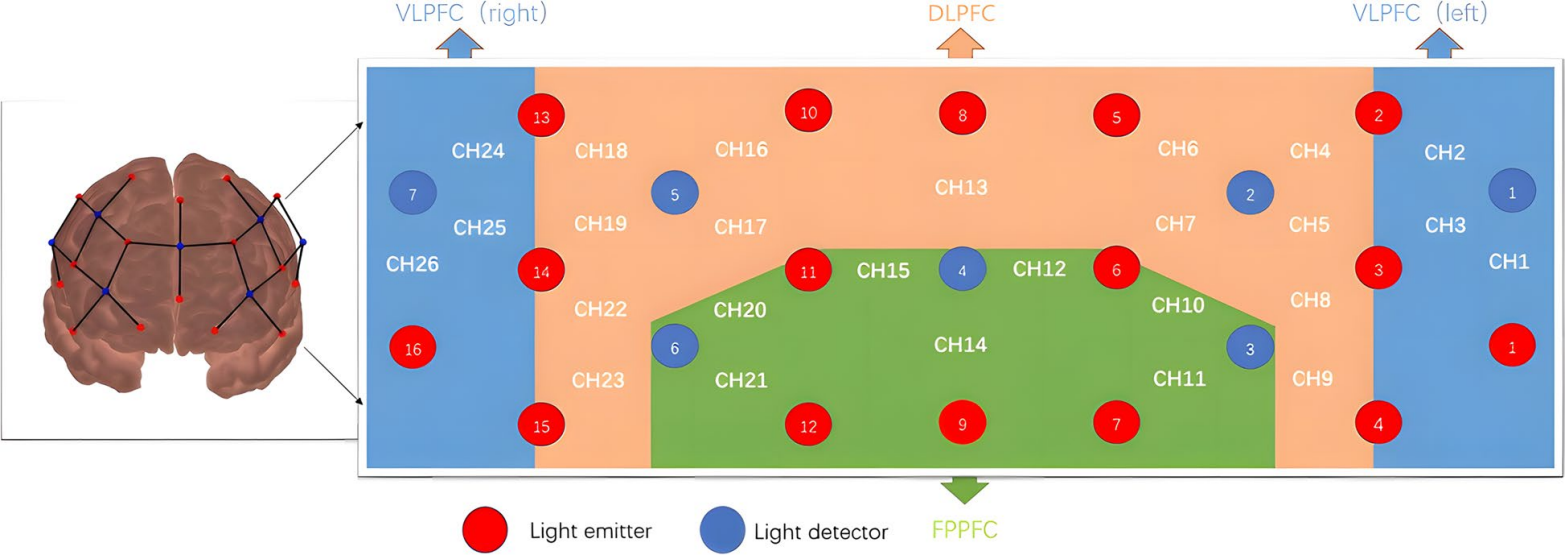
Probe locations and channel settings in 26-channel near-infrared spectroscopy. Brodmann area shows 26 sensory regions(from Ch1 to Ch26) within the prefrontal cortex

According to a previous study [30, 31] of anatomical craniocerebral correction via the international 10–20 system, a line is drawn between the fNIRS channel and the position of the cerebral cortex measurement. In accordance with the international 10–20 system, 26 channels are available: The following channels were available: According to Brodmann's area, channels 1–3, 24–26 are located in the ventrolateral PFC (VLPFC; BA44, 45, and 47), channels 10–12, 14, 15, 20, and 21 are located in the frontopolar PFC (FPPFC; BA 10). and channels 4–9, 13,16–19, 22–23 are located in the dorsolateral PFC (DLPFC;BA 9 and 46), based on Brodmann’s area [32] (Fig. 2).

### fNIRS Data analysis

fNIRS data can be analyzed with the toolbox homer2, which offers a graphical user interface based on MATLAB [33]. A band-pass filter operating between 0.01 and 0.1 Hz was used to remove high-frequency noise from the raw data. Following band-pass filtering, a threshold of 30 dB was used to locate the noise in the detection channel and eliminate slowly drifting noise from the body and environment [34]. Motion artifacts were then removed using a processing technique based on moving standard deviations and cubic spline interpolation [35, 36]. The sliding window's standard deviation over a predetermined threshold was used to locate artifacts. After that, cubic spline interpolation was used to eliminate them. Based on the filtered optical data, the [oxy-Hb] concentration was calculated using an improved version of the beer Lambert Law [37]. We focused on[oxy-Hb] since it provides a more accurate indication of cortical activity than [deoxy-Hb]. As a matter of fact, it is believed to have a stronger correlation with BOLD signals detected by fMRI and a stronger response to cognitive task-induced brain activation [38]. As the baseline, we took the last ten seconds of the pre-task break. Each channel contains the average [oxy-Hb] levels for each participant during the task period and at baseline. By deducting the baseline mean [oxy-Hb] value from the task period mean [oxy-Hb] value, the [oxy-Hb] value during the VFT was calculated. Through this, we were able to determine the mean difference between the baseline and task periods for [oxy-Hb].

### Statistics

Software SPSS version 26.0 was used to conduct the statistical analysis. The baseline demographics of the groups with MDD and HCs were analyzed via the chi-square test (sex) and the variance analysis(age & education). The total number of words uttered by each subject group was then compared via a variance analysis, multiple comparisons were performed on their significantly different results, and the HAMD-24, HAMD without sleep disorders,HAMA-14,PSQI score for each patient was compared via a t-test. the [oxy-Hb] values between the MDD and HCs groups during the task period for each channel during the VFT were compared via Kruskal–Wallis h tests in the following order. This analysis of our fNIRS data was conducted. via the Mann–Whitney test, the [oxy-Hb] values of the insomniacs and the non-insomniacs are compared. We conducted numerous comparisons of neural activation across different channels via a false discovery rate [39]. *p* < 0.05 was set as the significance level. Pearson correlation analysis of HAMD-24, HAMA-14, and PSQI scores with [oxy-Hb] values in each channel during the VFT in patients with MDD and HCs.

## Result

### Demographic and clinical characteristics

In terms of gender (chi-square test: *X2* = 2.345, *p* = 0.31), age (ANOVA: *F* = 0.391, *p* = 0.677), or level of education (ANOVA: *F* = 0.489, *p* = 0.614), MDD patients and HCs are not significantly different. Patients with insomnia scored higher on the HAMD-24, HAMA-14, and PSQI than those without insomnia. The HAMD-24 and PSQI scores differed significantly (t-test: *t* = -3.337, *p* = 0.001), but not the HAMA-14 scores (t-test: *t* = -1.855, *p* = 0.067)and HAMD without sleep disorders scores (t-test: *t* = 0.009, *p* = 0.993). In terms of the words on the VFT, HCs and MDD patients without insomnia performed similarly (LSD-t: *t* = 0.148, *p* = 0.876), but MDD patients with insomnia performed significantly worse than the other two groups (LSD-t: *t* = 3.333–3.481, *p* < 0.001). The study participants' demographic and medical data are shown in Table 1.

**Table 1:** Participants' demographic characteristics and clinical characteristics

	HCs	patients with MDD		× 2/F/t	P
		without insomnia	With insomnia		
n	44	32	48		
**Demographic**					
Age, years	27.89 ± 6.85	26.38 ± 6.70	26.94 ± 8.72	0.391	0.677
Gender, male/female, n	13/35	9/23	18/26	2.345	0.31
Education, years	14.68 ± 2.74	14.68 ± 2.74	14.17 ± 3.48	0.489	0.614
**Clincal**					
HAMA-14 scores	3.68 ± 2.23	21.13 ± 5.97	24.04 ± 7.43	1.855	0.067
HAMD-24 scores	3.80 ± 1.92	25.22 ± 6.91	31.10 ± 8.22	3.337	0.001
HAMD-24 without sleep disorder scores	3.16 ± 1.63	24.38 ± 6.79	24.36 ± 6.38	0.009	0.993
PSQI scores	6.95 ± 3.31	6.97 ± 2.32	13.15 ± 2.36	-11.542	0.001
Number of words, n	10.02 ± 4.59	9.88 ± 4.35	6.54 ± 3.27	10.444	0.001

### Characteristics of[oxy-Hb]Signals changes during the VFT

**Fig. 3:**
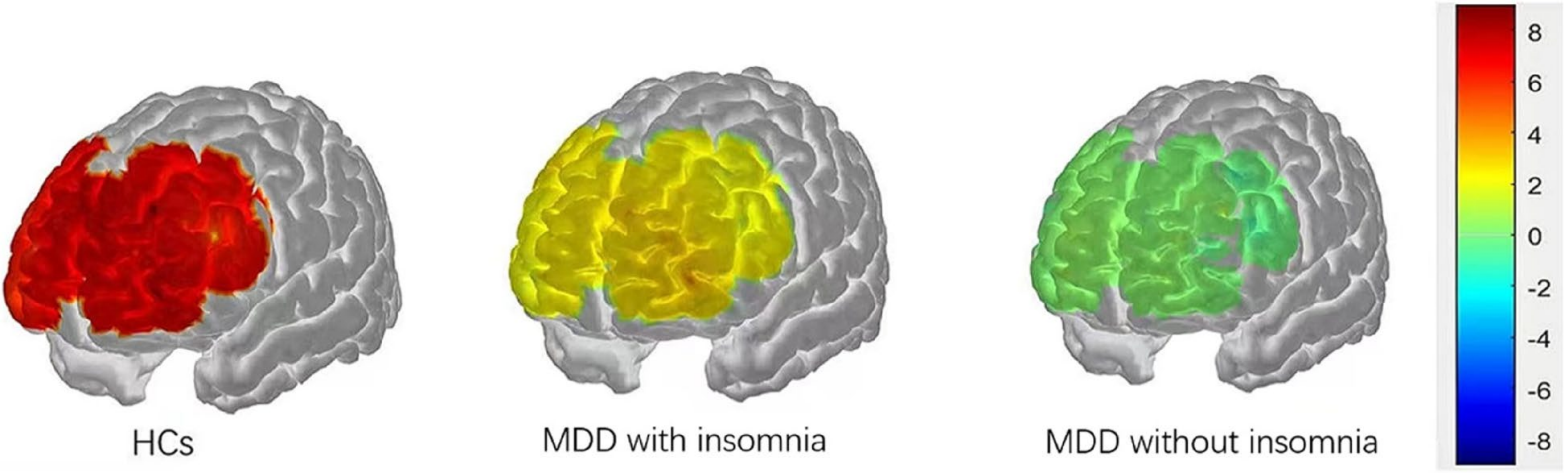
Prefrontal cortical activation during the VFT in patients with or without insomnia and HCs.The color scale depicts the change of [oxy-Hb] value range from -0.8 to 0.8 in umol x mm

**Fig. 4:**
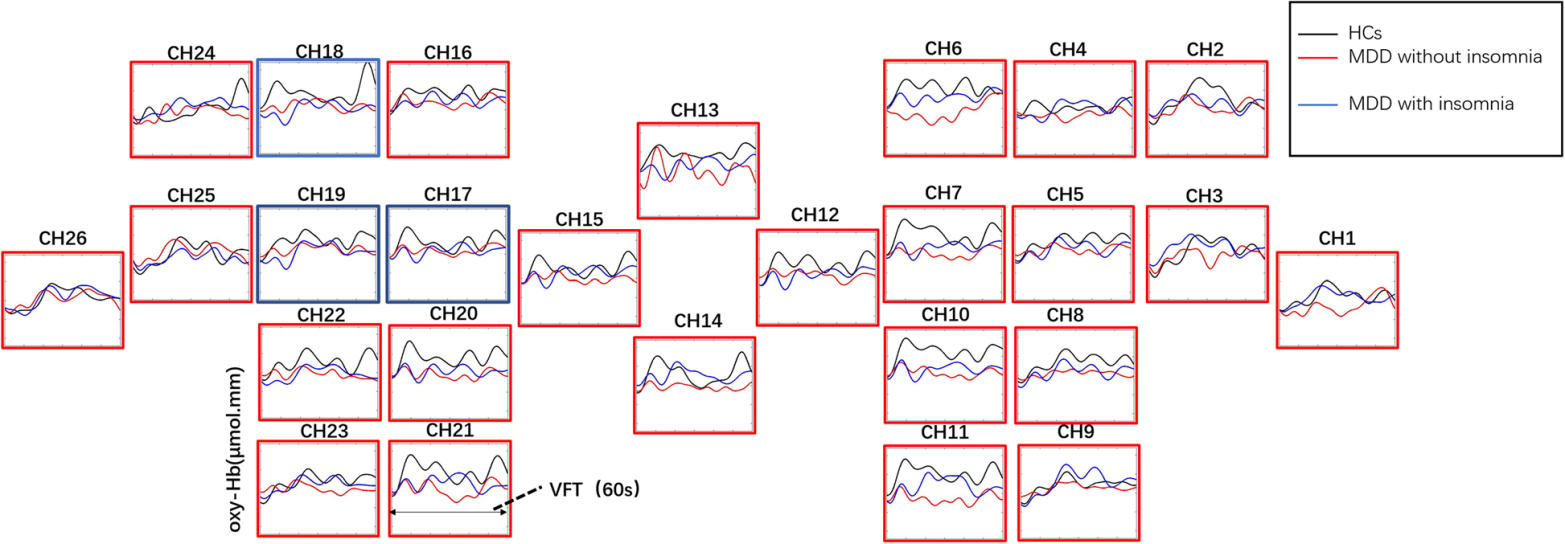
Waveforms of [oxy-Hb] values during the VET in the 26 channels over prefrontal regions in patients with insomnia and without insomnia and HCs. Red boxes indicate channels with significantly higher [oxy-Hb] values in the insomnia group than in the without insomnia group. Blue boxes indicate channels with significantly higher [oxy-Hb] values in the without insomnia group than in the insomnia group

The [oxy-Hb] values of HCs were found to be higher than those of the MDD group in all channels of the VFT task (Kruskal Wallis h test:H = 50.721 ~ 2305.357,* p* < 0.01). In the MDD group, [oxy-Hb] values were higher in the insomnia group than in the no insomnia group in Ch1-16, 20–23 (Mann Whitney test:z = 1.171 ~ 10.4197,FDR *p* < 0.01). And in Ch17-19, [oxy-Hb] values were higher in the no insomnia group than in the insomnia group (Mann Whitney test: z = -1.46 ~ -39.491, *p* =  < 0.001) (Figs. 3 and 4).

### Correlation Between fNIRS data and clinical data

**Fig. 5:**
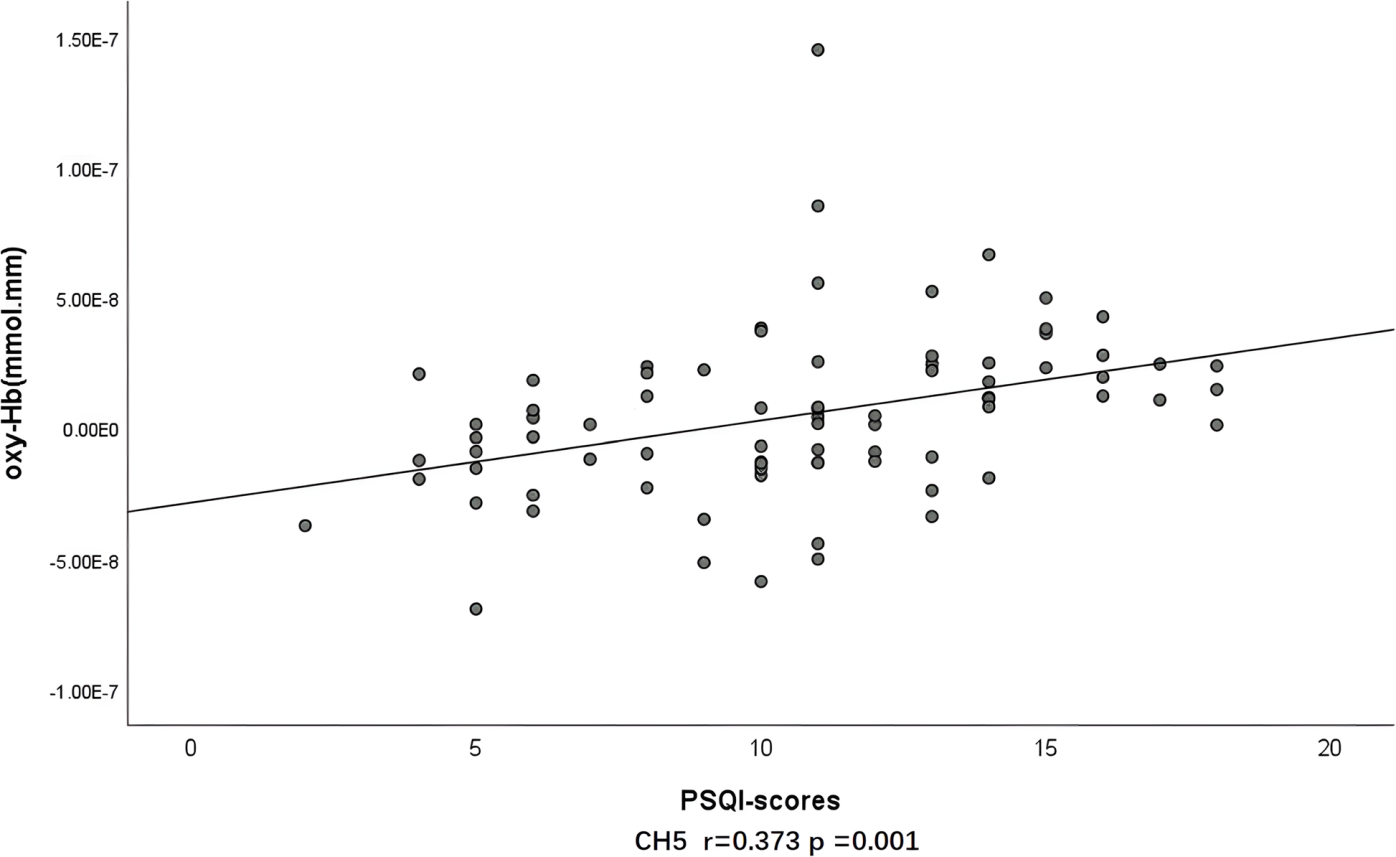
Correlation between PSQI scores and [oxy-Hb] values in CH5

HCs and MDD groups showed no relationship between total number of words uttered and [oxy-Hb] values in all channel; HAMA-14 scores and HAMD-24 scores were not related to [oxy-Hb] in all channel. There was no relationship between PSQI scores and [oxy-Hb] values in all channels in the HCs group. However, in the MDD group, the [oxy-Hb] values of channels 1–3, 5, and 7 were positively correlated with PSQI scores. (Pearson's *r* = 0.223–0.440, *p* = 0.01 ~ 0.048) (Fig. 5).

## Discussion

A study using fNIRS was conducted for the first time to explore the effect of insomnia on the level of activation of brain function in MDD patients. MDD patients with insomnia were significantly more activated in all brain regions except the right DLPFC compared to those without insomnia, However, insomniacs possessed a lower number of word production during VFT. And patients' activation levels were positively correlated with the severity of insomnia in some left brain regions, but not with the severity of depression or anxiety.

Previous fNIRS studies of the VFT have shown that MDD patients have decreased PFC activity compared with healthy individuals [13, 40, 41]. In comparison to HCs, patients with MDD had significantly lower blood flow in the bilateral DLPFC (BA 9, 46), VLPFC (BA 44, 45, 47), and FPPFC (BA 10) during the VFT [42]. Our findings are consistent with those of previous studies, which also discovered that various PFC regions (bilateral FPPFC, DLPFC, and VLPFC) were less active. Patients with MDD were less active during the VFT, which suggests that their PFC function is low on both sides. PFC activity during VFT is lower in MDD patients than in healthy controls, particularly on the left side, according to a number of fMRI studies [43, 44]. This might be brought on by neuronal dysfunction brought on by neurovascular coupling mechanisms [45] or by decreased cerebral blood flow [46].

In accordance with earlier studies [47, 48], we found no significant correlation between HAMD and HAMA scores and oxy-Hb levels. It has been shown that HAMD scores and oxy-Hb levels on fNIRS are correlated with depressive symptoms in some studies [17, 49, 50], but not in others. The variations in these findings could be attributed to the different patient populations and methods used by each study to measure changes in [oxy-Hb].

Guillermo Borragán and his colleagues found that normal people who went without sleep for a short time had more PFC activity in the right prefrontal cortex and less PFC activity in the left brain [18]. According to Motoyasu Honma et al., activation of the right prefrontal cortex (but not the left prefrontal cortex) correlates positively with alertness during an alertness test in healthy subjects. Therefore, activation of the right prefrontal cortex may assist healthy individuals or people with short-term insomnia in overcoming sleepiness and may also provide sufficient activity to meet the cognitive demands of higher cognitive tasks [19].

Do chronic insomniacs and MDD patients overcome sleepiness in the same brain regions as healthy individuals? According to Sun et al., chronic insomniacs perform verbal tasks with significantly lower PFC activation than normal subjects. Oxy-Hb changes in some left prefrontal cortex tend to increase progressively with decreasing sleep quality [11]. In our study, we found that MDD patients with insomnia had higher activation in the left hemisphere with lower sleep quality, similar to "chronic insomniacs who are typically more activated in the left hemisphere during VFT, in contrast to healthy individuals. However, although both chronic insomniacs and MDD patients confront insomnia in the left side of the brain, the sites are different, with MDD patients closer to the VLPFC and chronic insomniacs closer to the orbitofrontal cortex.In conclusion, the brain areas in which chronic insomniacs, MDD patients, healthy people and individuals with short-term insomnia overcome sleepiness differ. Previous studies have found extensive bilateral brain atrophy in patients with major depression and chronic insomnia, for example, the orbitofrontal cortex (OFC), middle prefrontal cortex and anterior cingulate cortex (ACC) in patients with depression have smaller gray matter volumes [51], and the frontal parietal lobe and ACC in patients with chronic insomnia have smaller gray matter volumes [52]. At the same time, we noticed that the atrophy of Brodmann area 24, anterior cingulate cortex, medial orbital and other areas in depressed patients was more severe in the right brain than in the left brain [53, 54]. However, patients with chronic insomnia also have more severe shrinkage of the right DLPFC volume [55]. So we hypothesized that the likely reason why healthy subjects overcame the sleep brain regions differently than depressed and chronic insomnia subjects may be due to more pronounced damage to the right side of the brain in both chronic insomnia and depression subjects. However, we do not know why chronic insomniacs are different from those with MDD, and more research is needed to explore.

So, Can activation of the left prefrontal cortex provide sufficient activity for chronic insomniacs and MDD patients to meet the demands of higher cognitive tasks?In our experiments, unlike chronic insomniacs who did not differ significantly in the number of words produced relative to healthy individuals, MDD patients with insomnia produced significantly fewer words than MDD patients without insomnia, which we assume is related to impaired left prefrontal function in MDD patients.

In light of the fact that many studies evaluating efficacy (e.g., pharmacological or transcranial magnetic stimulation) have not considered insomnia as a factor in activation [56, 57], we recommend that sleep metrics be included in future cross-sectional comparisons of MDD patients.

Our study has some limitations. First, our fNIRS signal in a typical source-detector channel may be contaminated with systematic interference in the head surface [58]. The use of additional short source-detector separation optodes was studied to eliminate systematic interference and improve the accuracy of fNIRS measurements [59]. Second, in the present study, we are not sure what factors will significantly influence activation, and this study, although excluding the influence of drugs, may still be influenced by other factors (such as duration of illness and the number of depressive episodes).

## Conclusions

The PFC brain region was significantly less active during VFT in those with MDD than in healthy controls. All brain regions except for the right DLPFC showed significantly higher activity in MDD patients with insomnia compared to those without insomnia, suggesting that sleep quality needs to be an important indicator in fNIRS screening. Furthermore, there was a positive correlation between the severity of insomnia and left VLPFC activation levels, indicating that the left brain region, particularly the VLPFC, plays a crucial role in overcoming sleepiness in MDD patients; these findings may provide new ideas for the treatment of insomnia in MDD patients in the future. 

## Data Availability

The datasets used and/or analyzed during the current study are available from the corresponding author on reasonable request.
